# Segmental hair analysis as a retrospective testosterone diary: possibilities and pitfalls

**DOI:** 10.1038/s41598-023-41672-7

**Published:** 2023-09-25

**Authors:** Julia K. Preinbergs, Jakob O. Ström, Elvar Theodorsson, Edvin Ingberg

**Affiliations:** 1 Department of Biomedical and Clinical Sciences, Division of Clinical Chemistry and Pharmacology, Linköping University, Linköping, Sweden; 2 Department of Neurology, Faculty of Medicine and Health, Örebro University, Örebro, Sweden; 3 Department of Infectious Diseases, Faculty of Medicine and Health, Örebro University, Örebro, Sweden

**Keywords:** Steroid hormones, Immunochemistry, Endocrine system and metabolic diseases, Predictive markers

## Abstract

Testosterone is thought to be incorporated in growing hair strands so that specific hair segments reflect average free hormone concentrations from the corresponding time period. However, the exact mechanisms of hormone integration in scalp hair have not yet been established and it is not known how testosterone is stored in the hair segments over time. The aim of this study was to investigate the stability of testosterone concentrations in hair as it grows and to determine if segmental hair analysis can be used as a retrospective testosterone diary. Thirty men and 40 women provided two hair samples and 16 saliva samples during a period of three months. Hair growth between the two samplings was measured. Hair samples were cut into 10 mm segments resulting in three segments from the first sampling and six segments from the second sampling. Hair samples were pulverised and extracted with methanol. Hair testosterone concentrations were analysed using an in-house radioimmunoassay. Salivary testosterone was analysed using a commercial enzyme-linked immunosorbent assay (Demeditec). The results demonstrated that there is a degree of segmental hormone conservation over time (rho = 0.405–0.461, *p* < 0.001, n = 66–67), but also highlighted three potential confounders. Firstly, testosterone concentrations were higher in distal hair segments (mean concentration ratio most distal by most scalp-near was 1.55, SD 0.70), which may be due to continuous hormone integration from sebum and changes in hair matrix composition. Secondly, more frequent hair washing stunted the increase in testosterone concentrations in distal segments (rho = −0.404, *p* =  < 0.001, n = 66). And lastly, intra- and inter- individual variability in hair growth rate influenced the temporal resolution along the hair, although mean growth rate was indeed 30.0 mm for three months. In a multiple regression model the biological sex, natural hair colour, and relationship status were significant explanatory variables to hair testosterone concentrations. The current results indicate that repeated hair sampling near the hair roots during a study may be preferable to analysing concentration changes between proximal and distal segments within the same hair sample. Also, hair testosterone analysis needs to be adjusted for sex and the natural hair colour.

## Introduction

Analysis of endogenous steroid hormones in hair has gained popularity during recent decades, with primarily cortisol being evaluated due to its association with long-term biological and/or mental stress^1,2^. The utility and biological implications of hair testosterone concentrations have been investigated to a much lesser degree. An average growth rate of scalp hair of 1 cm per month has been the foundation for a hypothesis that a certain hair segment reflects average free endogenous hormone concentrations during a specific retrospective period^3,4^. The potential of segmental hair hormone analysis as a retrospective diary has been examined thoroughly regarding cortisol with somewhat conflicting results. Some research groups have reported distinctly declining cortisol concentrations with increased distance from the scalp, while others have reported stable concentrations and even pointed out correlations in time between pathological hair hormone levels in a certain hair segment and a clinical diagnosis or treatment^5,6^. Two obvious differing factors are the choice of the analytical method (immunoassay or liquid-chromatography tandem mass spectrometry) and whether a pre-extraction decontaminating wash is performed. Interestingly, Dettenborn et al.^7^ mentioned an unpublished comparison where omitting the pre-extraction wash eliminated the problem of decreasing cortisol concentrations in distal hair segments. Cortisol in hair has also been shown to decrease with exposure to ultra violet (UV) light and various hair treatments, and it remains unclear whether this is due to decreased hormone content or changes in the hair matrix with increasing distance from the scalp^8–10^. For testosterone in hair the segmental stability in hormone concentrations has not yet been systematically studied. Also, the effects of hair washing frequency (i.e. hair washing at home) have been shown to significantly decrease cortisol concentrations in distal hair segments, but this has not yet been evaluated for hair testosterone^7^.

Mass spectrometric methods for hair testosterone analysis have evolved over the last years, and have to some extent replaced immunoassays for this purpose. However, mass spectrometric methods still present with some difficulties regarding detection limits, particularly in hair samples from women^11,12^. The radioimmunoassay used in this study has proven to reliably measure testosterone in hair from both men and women^13,14^. No immunoassay is perfectly selective, but we have previously shown with chromatography that the majority of the immunoreactivity consisted of testosterone^13^.

The aim of this study was to investigate the stability of testosterone concentrations in hair as it grows and to determine if segmental hair analysis can be used as a retrospective testosterone diary. Hair samples were cut three months apart as close to the scalp as possible and the hair locks were then cut into 10-mm-long segments. Assuming uniform hair growth at a rate of 1 cm per month, the first hair sample (consisting of three segments) and the distal half of the second hair sample represented the same time period. Furthermore, the correlation between hormone levels in hair and saliva was assessed as well as the performance of both hair and saliva to differentiate between men and women based on mean testosterone concentrations. The stability of testosterone concentrations within segments with increasing distance from the scalp was evaluated without a pre-extraction decontaminating wash, to avoid the risk of confounding from increased wash-out of lipophilic substances in distal hair segments. The effect of the study participants’ hair washing habits on the stability of hair hormone concentrations was evaluated, and the contribution of other confounding factors was explored. Finally, the individual rate of hair growth during 3 months was measured in regrown hair in the first hair sampling area.

## Materials and methods

### Participants

Participants were 30 men and 40 women recruited via posters on public information boards located at the university campuses and in the hospitals in three cities (Linköping, Norrköping and Örebro) in Sweden. All participants gave written informed consent prior to inclusion. The study was approved by the Regional Ethical Review Board, EPN 2013/100-31, Linköping, Sweden. The study was performed in accordance with relevant guidelines and regulations. Information on age, sex, body weight and length, natural hair colour, current medication, hair wash frequency, hair heat treatment frequency, education level, occupation, social status, and the number and age of any biological children was recorded at inclusion, see Table 1.

**Table 1:** Baseline data.

	Men n = 30	Women n = 40
Age (mean, years)	32.2 (SD 12.3) Min–max 18–67	36.3 (SD 12.9) Min–max 19–77
BMI (mean, kg/m^2^)	23.6 (SD 2.3)	23.7 (SD 3.8)
Relationship status, n (%)		
Single	9 (30)	10 (25)
Partner (not cohabitating)	5 (16.7)	5 (12.5)
Partner (cohabitating)	16 (53.3)	25 (62.5)
Education level*, n (%)		
Compulsory school (EQF level 3)	0 (0)	1 (2.5)
Upper secondary (EQF level 4)	10 (33.3)	8 (20)
Post upper secondary education (EQF level 5)	1 (3.3)	7 (17.5)
Bachelor’s degree (EQF level 6)	5 (16.7)	6 (15)
Master’s degree (EQF level 7)	14 (46.7)	17 (42.5)
Doctorate (EQF level 8)	0 (0)	1 (2.5)
Occupation, n (%)		
Student	13 (43.3)	13 (32.5)
Unemployed	1 (3.3)	0 (0)
Employed	15 (50)	26 (65)
Pensioner	1 (3.3)	1 (2.5)
Biological children, n (%)		
None	17 (56.7)	19 (47.5)
Younger than 18 years	11 (36.7)	16 (40)
Older than 18 years	2 (6.7)	5 (12.5)
Alcohol intake**		
None, n (%)	8 (26.7)	15 (37.5)
Units per week	Median 2.0 IQR 3.0	Median 1.0 IQR 1.5
	Min–max 1.0–10.0	Min–max 0.5–8.0
Natural hair colour, n (%)		
Light	6 (20)	7 (17.5)
Medium	13 (43.3)	23 (57.5)
Dark	8 (26.6)	7 (17.5)
Red	2 (6.6)	1 (2.5)
Grey	1 (3.3)	2 (5)
Hair washes per week, n (%)		
0	1 (3.3)	1 (2.5)
1	0 (0)	3 (7.5)
2	6 (20)	15 (37.5)
3	10 (33.3)	14 (35)
4	3 (10)	5 (12.5)
5	1 (3.3)	0 (0)
6	2 (6.7)	2 (5)
7	7 (23.3)	0 (0)
Hair heat treatment per week, n (%)		
0	28 (93.3)	32 (80)
1	1 (3.3)	6 (15)
2	0 (0)	1 (2.5)
3	0 (0)	1 (2.5)
4	0 (0)	0 (0)
5	1 (3.3)	0 (0)

*Education level is the highest completed level, each category specified in accordance to the European Qualifications Framework (EQF).

**One standard unit is equivalent of 12 g ethanol.

Inclusion criteria:

- Age 18 or older
- Scalp hair length of minimum three centimetres at the posterior vertex

Exclusion criteria:

- Tobacco use
- Hair colouring or bleaching during the previous six months
- Planned significant change in daily life (such as training for a competition) during the study
- Chemical or surgical castration
- Hormonal treatment intended for sex change/gender reassignment or treatment for hypo-/hypercortisolism or hypogonadism
- Pregnancy or fertility treatment during the previous six months or plans for pregnancy during the study
- Intake of anabolic steroids or other narcotics during the previous six months or planned intake of such substances during the study

Intake of oral contraceptives was allowed. The participants agreed to not cut their hair during the study.

During the first visit the technique of at-home saliva sampling was demonstrated, and written instructions were also given. The participants were asked to abstain from drinking, eating or brushing their teeth for a minimum of one hour before the sampling and to put the saliva sample immediately in the home freezer. A total of sixteen saliva samples per person were collected for four weeks. Each participant chose two optional days during each sampling week and collected one saliva sample in the morning and one saliva sample in the evening. Samples were collected between 6.00 and 9.00 AM and PM. If a participant forgot to take a saliva sample on a planned sampling day it was allowed to try to complete a new set of morning and evening sampling on another day during the same week. The samples were brought by each participant to the final visit in a cool box with ice packs to keep the samples from thawing during transport, and stored at  − 20 °C until the day of analysis.

Hair samples were cut at the posterior vertex as close to the scalp as possible. The area of the sampling was approximately 2.5 × 0.5 cm. At the second visit three months later, the posterior vertex of the participants was combed through, identifying the sampling area from the first hair sampling. The regrown hairs were of differing lengths, resembling an ink brush. It was not possible to measure the regrown length of the hair from the first sampling with only one measurement and therefore the shortest and the longest hairs in the first hair sampling area was measured in millimetres using a transparent ruler. The second hair sample was then cut as close to the scalp as possible in an adjacent scalp area at the posterior vertex.

### Testosterone analysis

**Figure 1:**
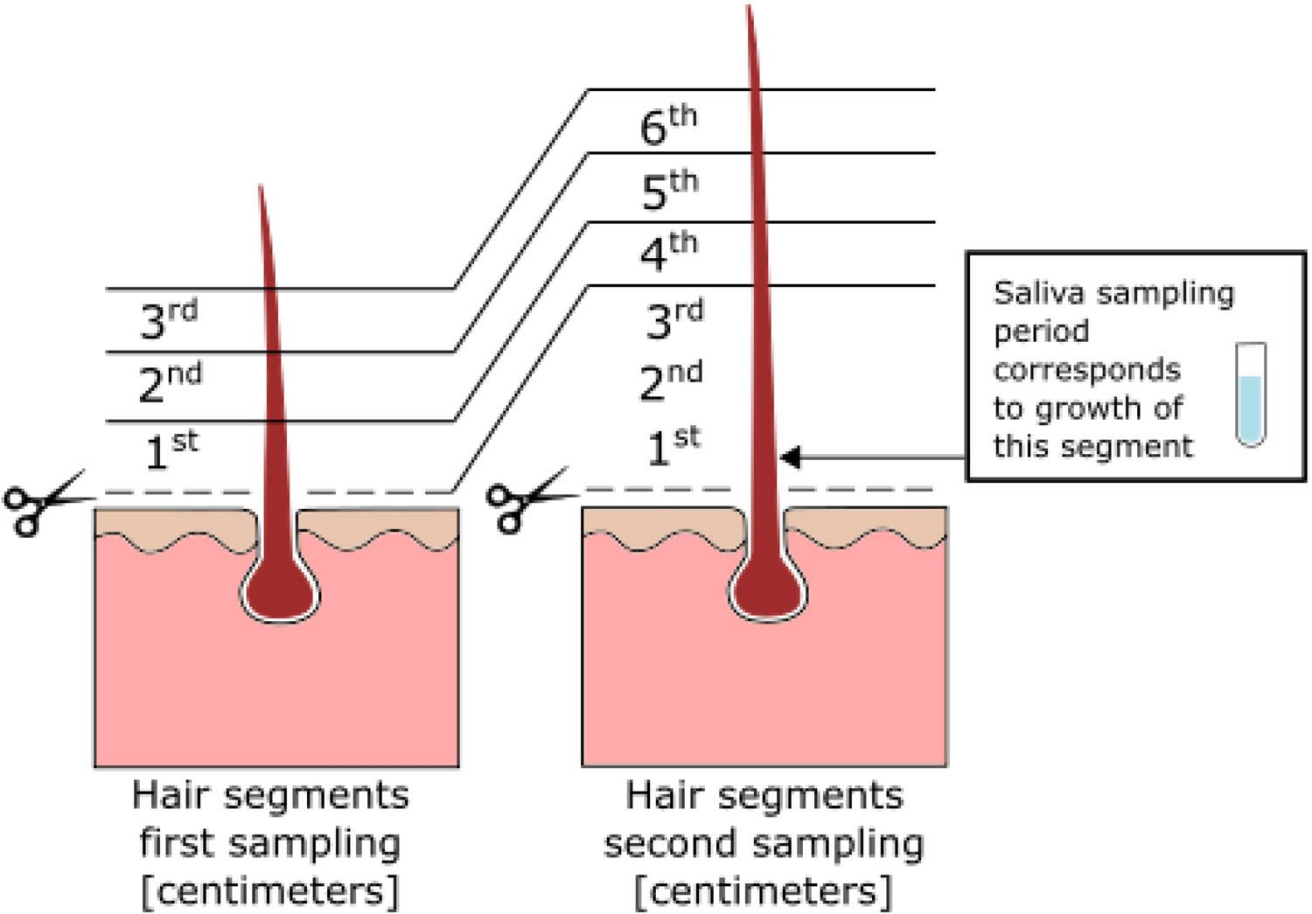
Outline of the hair segments from the two samplings (three months apart) and timing of the saliva sampling period. The three hair segments from the first sampling would correspond to the distal three segments from the second hair sampling based on an average hair growth rate of 1 cm per month.

The most proximal three centimetres of the first hair sample and the most proximal six centimetres of the second hair sample were analysed. The hair was divided into 10 mm long segments (three segments from the first hair sample and six segments from the second hair sample), and the second hair sample was timed so that the distal three segments would, theoretically, correspond time-wise to the three segments of the first hair sample, see Fig. 1. Each hair segment was pulverised and extracted with methanol. Hair testosterone was measured using an in-house competitive radioimmunoassay in vacuum-concentrated extracts as previously described^13^.

Sample weights were approximately 5–10 mg, and only a few of the distal segments from males weighed less than 5 mg (n = 24, i.e. 3.9% of all hair segments in the study, including one sample that weighed less than 1 mg and was excluded from the analysis). All hair samples were analysed in duplicate in the same assay and all hormone measurements were above the limit of detection.

The salivary testosterone was analysed using a commercial enzyme-linked immunosorbent assay (ELISA) from Demeditec Diagnostics GmbH, Kiel, Germany (testosterone DES6622). Controls for low and high concentrations were supplied. All assays were from the same batch. Inter-assay variation for testosterone was 10% at low concentrations (mean 43.5 pg/mL) and 6% at high concentrations (mean 205 pg/mL). The saliva sampling period was timed for each participant so that the saliva samples would, theoretically, correspond timewise to the first segment of the second hair sample (see Fig. 1), with a two-week delay between the end of the saliva sampling period and the second hair sampling to account for the distance between the follicle and the surface of the skin^15^.

### Statistical analysis

Level of significance for all statistical analyses was set at α = 0.05, and tests were two-sided. The distribution of hair testosterone results was positively skewed. The distal three hair segments from the second sampling represented the same time period as the three most proximal segments from the first sampling as illustrated in Fig. 1. The segmental stability of hair testosterone concentrations was explored with Spearman correlation by calculating ratios between adjacent hair segments in the first hair sampling and comparing to the ratio for the corresponding hair segments from the second hair sampling. The correlation in hormone concentrations between matrices (saliva compared to hair) was evaluated with Spearman correlation with the average of both morning and evening saliva samples (n = 16 for each individual) compared to the first hair segment from the second hair sampling, which was timed to theoretically represent the same month when saliva samples were collected. Area under the curve statistics was used to test the ability to discriminate between sexes based on average testosterone concentrations in saliva and hair, respectively. The average of the most proximal hair segment from both samplings (termed mean individual “baseline” hair testosterone concentration), was used for this calculation in order to reduce measurement error. Related-Samples Wilcoxon Signed Rank Test was used to evaluate the intraindividual change in hair hormone concentrations between the most proximal and most distal hair segment from the second sampling. In order to test whether hair wash frequency had a cumulative effect on hormone concentrations in distal hair segments compared to proximal hair segments, a ratio between the most distal and most proximal hair segment (from the second hair sampling) was calculated and the effect of hair wash frequency assessed with Spearman correlation. Multiple linear regression was used to explore the effect of different background factors on hair testosterone concentrations. The average of the most proximal hair segments from both samplings was used (the mean individual “baseline” hair testosterone concentration) as the dependent variable. Possible explanatory factors were: biological sex (man/woman), age (in years, continuous variable), BMI (kg/m^2^), natural hair colour (light, medium, dark), hair wash frequency (times per week), alcohol consumption (standard units per week), children < 18 years (yes/no) and social status in dummy variables (single, not cohabitating with partner, cohabitating with partner). Regarding natural hair colour only three categories were used, thus, two persons with light red hair were transferred to the “light” category and one person with dark red hair was transferred to the “medium” category. Three individuals with grey hair did not contribute data to the hair colour variable in the regression analysis. Explanatory variables were chosen based on Spearman correlations with a cut-off of *p* ≤ 0.3 and included in the regression with forced entry. The robustness of the regression model was controlled by repeating it with transformed hair testosterone concentrations using the natural logarithm.

### Ethics approval and consent to participate

All participants gave written informed consent prior to inclusion. The study was approved by the Regional Ethical Review Board, EPN 2013/100-31, Linköping, Sweden.

## Results

### Stability between adjacent hair segments

**Figure 2:**
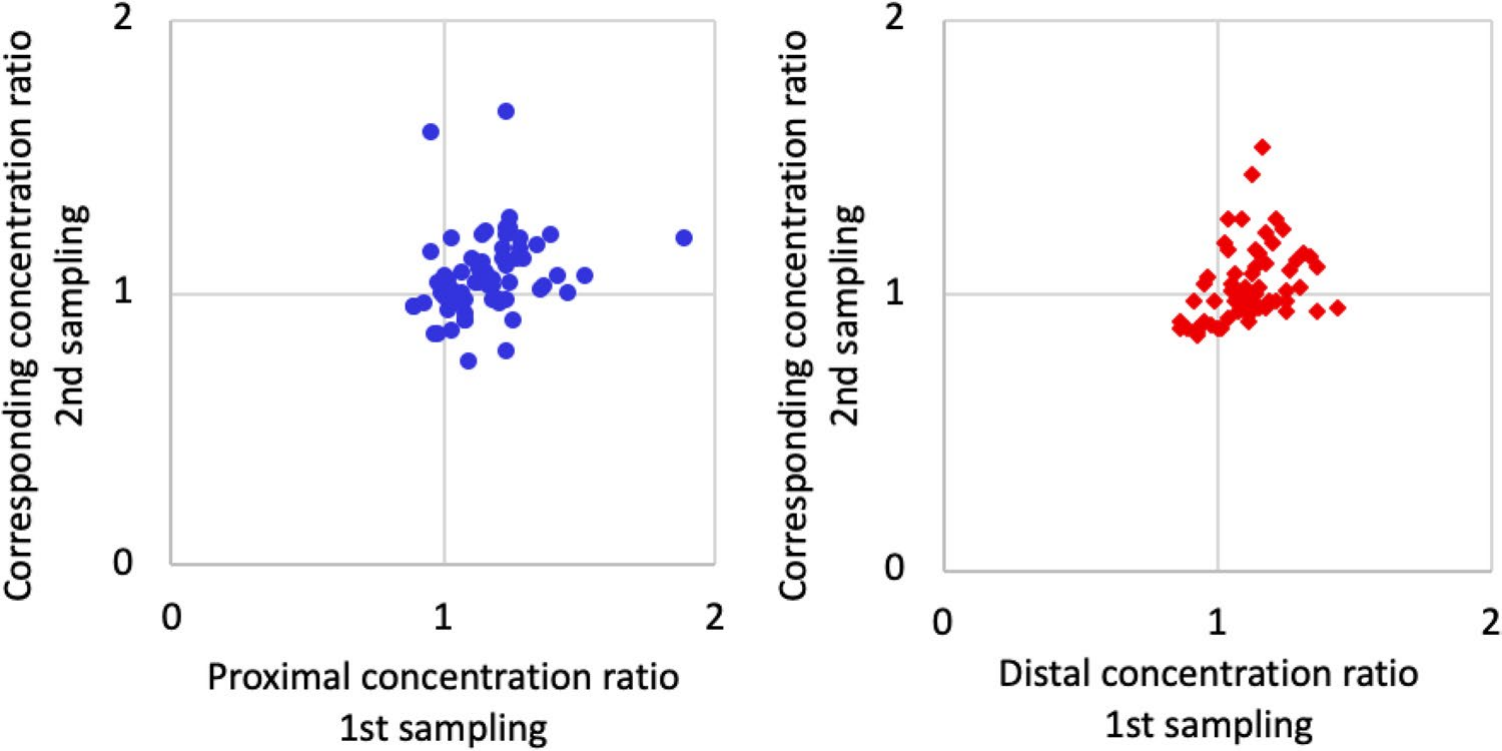
Concentration ratios describing the change in testosterone concentrations between adjacent hair segments compared to the corresponding segments (hair that has continued to grow on the scalp) from the second hair sampling. Blue dots show the ratio of the second and first segment from the first sampling compared to the ratio of the fifth and fourth segment from the second sampling (rho = 0.405, *p* < 0.001, n = 67). Red rhombi show the ratio of the third and second segment from the first sampling compared to the ratio of the sixth and fifth segment from the second sampling (rho = 0.461, *p* < 0.001, n = 66).

There was a degree of preservation of testosterone concentrations in the hair segments as the hair grew, but only to a moderate degree. To explore whether testosterone patterns along the length of the hair were preserved as the hair grew, we compared the relation between two adjacent hair segments in the first sample with the corresponding two segments from the second sample. The ratio between the first and second segment from the first sampling was compared to the ratio between the fourth and fifth segment from the second sampling, and the ratio between the second and third segment from the first sampling was compared to the ratio between the fifth and sixth segment from the second sampling. Spearman correlations were significant, but only of moderate strength (rho = 0.405–0.461, *p* < 0.001, n = 66–67; Fig. 2).

### Salivary testosterone in relation to hair testosterone and sex

**Figure 3:**
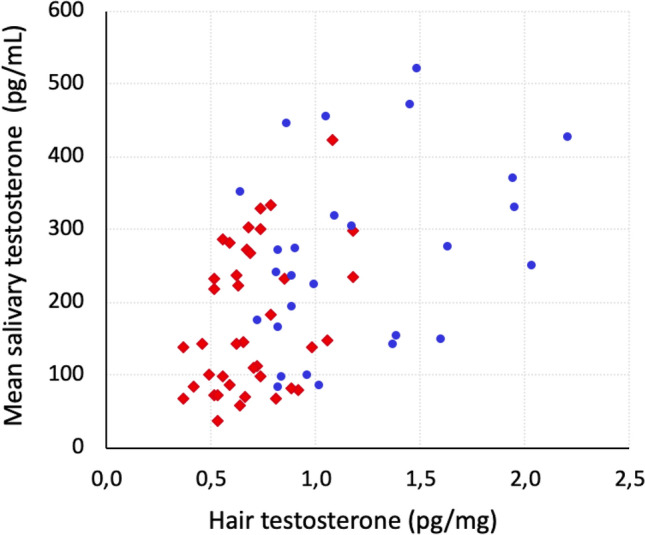
Scatter plot of hair testosterone concentration from the first segment from the second hair sampling and mean salivary testosterone (averaged from 16 saliva samples during one months’ time). Spearman correlation rho = 0.454, *p* < 0.001, n = 66. Women’s values are shown in red rhombi and the men’s values are shown in blue dots.

Testosterone concentrations in hair and mean saliva concentrations during the corresponding time period correlated to a moderate degree (rho = 0.454, *p* < 0.001, n = 66), see Fig. 3.

Testosterone concentrations in both saliva and hair reliably discriminated between men and women, but hair analysis was significantly superior in this regard. Mean salivary testosterone rendered a receiver operating characteristic (ROC) area under the curve of 0.718 (95% CI 0.595–0.841, *p* = 0.003), while mean baseline hair testosterone (averaged from the proximal segment from both samplings) produced a ROC area under the curve of 0.900 (95% CI 0.829–0.971, *p* = 0.000). Ad hoc, we tested if these confidence intervals overlapped according to Goldstein and Healy^16^, and found that the two areas under the curves were significantly different.

Saliva was collected within the specified time frame of 6.00–9.00 AM and PM in 96.8% of samples. The measurement range was exceeded in four out of 1043 saliva samples (from three men), and those samples were diluted and re-analysed. Three samples out of 1043 were below the limit of quantification and these came from one woman.

### Hormone concentrations in distal hair segments and effect of hair wash frequency

**Figure 4:**
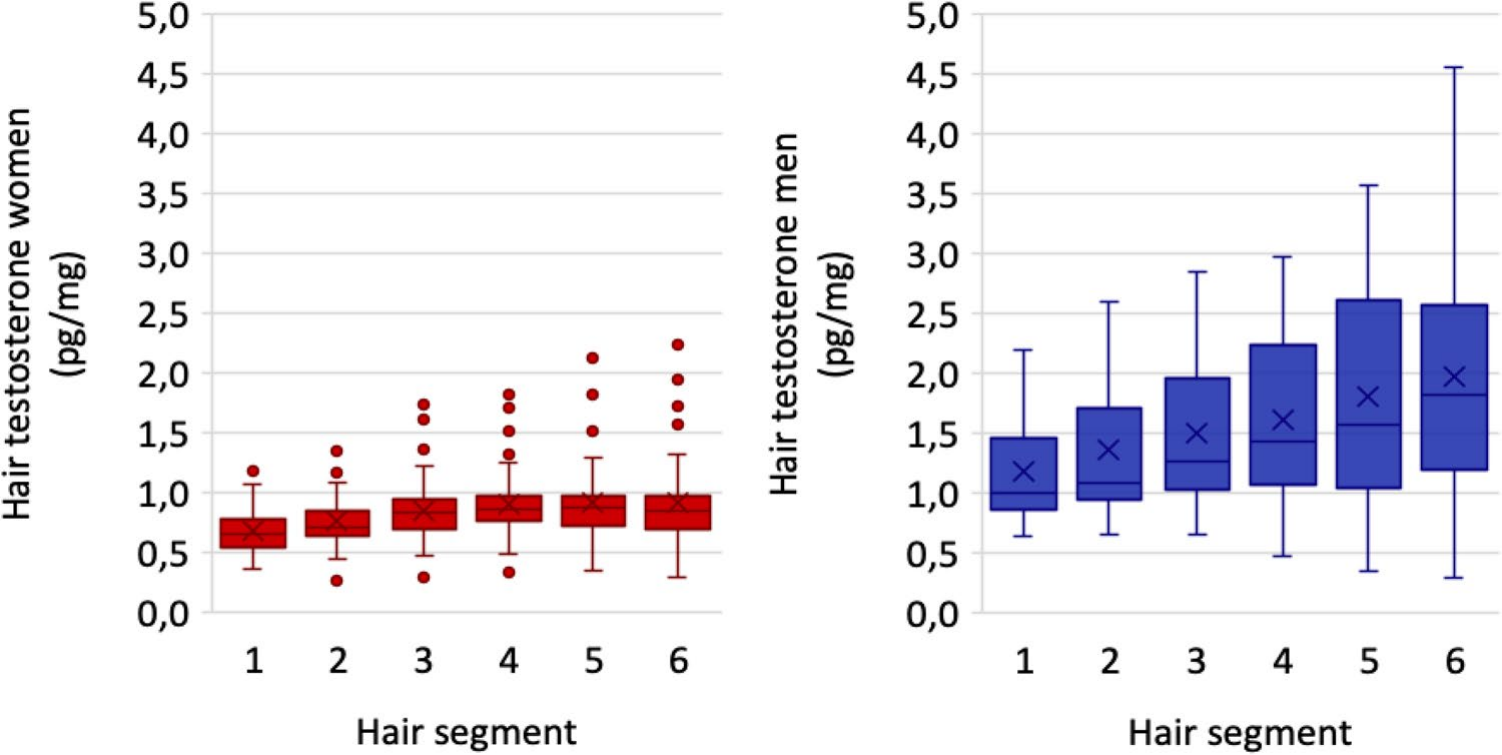
Hair testosterone in hair segments from the second hair sampling ranging from most proximal to most distal (segment 1 to 6). Mean concentration ratio was 1.55, SD 0.70, indicating higher concentrations in segment 6 compared to segment 1. In the figure, one male outlier with concentration 7.99 pg/mg in segment 6 has been excluded.

**Figure 5:**
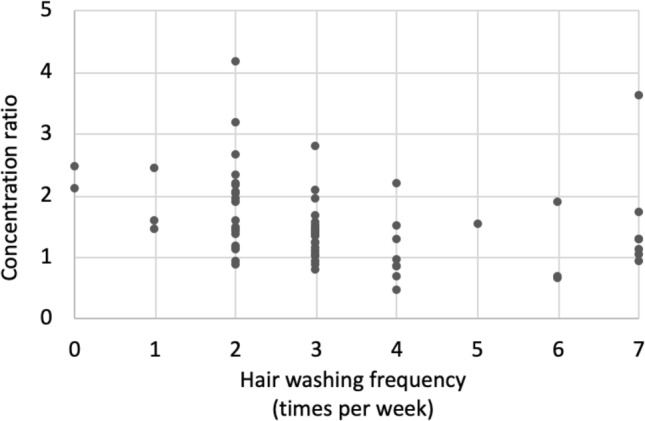
Concentration ratio (of the most distal by most proximal hair segment from the second hair sampling) and hair wash frequency. With more frequent hair washing habits the increase in distal testosterone concentrations became less pronounced (rho = −0.404, *p* =  < 0.001, n = 66).

Testosterone concentrations increased from proximal (i.e. closest to the scalp) to distal hair segments (Wilcoxon between most proximal and most distal segments from the second sampling; *p* < 0.001), see Fig. 4. In order to describe the change in concentration in each individual, a ratio between the most distal and most proximal hair segment from the second sampling was calculated. The more often the participants washed their hair, the less pronounced was the distal increase in hormone concentrations (rho = −0.404, *p* =  < 0.001, n = 66), see Fig. 5.

### Baseline hair hormone concentrations and explanatory factors

Explanatory variables included in the model were: biological sex, natural hair colour, hair wash frequency, relationship status as dummy variables and also BMI because of the plausible biological connection between body composition and testosterone concentrations as well as a univariate *p *value of close to 0.3 (Table 2). In the multiple regression for mean baseline hair testosterone the predictor variables sex, natural hair colour and cohabitating with a partner remained statistically significant (model R = 0.809, R^2^ = 0.655, *p* =  < 0.001), see Table 2. Repeating the regression with transformed mean baseline testosterone (using the natural logarithm) did not change the model fit or statistical significance.

**Table 2:** Factors associated with mean baseline hair testosterone.

	Univariable analysis		Median comparison^a^	Multivariable analysis			
	rho	*p* value	*p *value	*B*	*SE B*	ß	*p* value
Constant				.345	.282		
Biological sex	**.686**	** < .001**	** < .001**	**.437**	**.070**	**.529**	** < .001**
Age	**− .274**	**.022**	**.007**^**b**^	− .004	.003	− .101	.228
BMI	− .117	.333	**.090**^**c**^	.003	.010	.024	.760
Natural hair colour	**.326**	**.007**	**.019**	**.248**	**.048**	**.406**	** < .001**
Hair wash frequency	**.266**	**.026**	.059^d^	.017	.020	.073	.390
Children < 18 years	− .068	.579	.575				
Alcohol consumption	.025	.835	.915^e^				
Relationship status (partner, not cohabitating)	**.242**	**.043**	**.044**	.174	.101	.152	.091
Relationship status (partner, cohabitating)	**− .306**	**.010**	**.011**	**− .187**	**.077**	**− .225**	**.019**

Univariable Spearman correlation coefficients (n = 70 except natural hair colour where n = 67). Multiple regression model with forced entry of explanatory variables, model R = .809, R^2^ = .655, *p* =  < .001. *B*: unstandardized beta. *SE B*: standard error for the unstandardized beta. ß: standardized beta.

Significant values are in bold.

^a^Mann-Whitney U test, or Independent-samples Kruskal–Wallis, where applicable.

^b^Age dichotomised, ≤ 40 years or > 40 years.

^c^BMI dichotomised, < 25 or ≥ 25 kg/m^2^.

^d^Hair wash frequency dichotomised, ≤ 3 or > 3 washes per week.

^e^Alcohol consumption dichotomised, any consumption, or no consumption at all.

### Variation in hair growth rate

To illustrate intra-individual variation in hair regrowth, the shortest and the longest regrown hairs from the first hair sampling area were measured during the second hair sampling, which was three months after the first sampling. Only hairs with clear cut ends were measured. Mean hair growth was 30.0 mm (SD 3.9 mm, n = 57) during three months, which can be translated to an average growth rate of 10 mm per month. The within-individual difference between the shortest and the longest hairs in the regrown hair lock was on average 12 mm (min 3 mm, max 23 mm) per three months, which corresponded to 40% of the average outgrown hair length during the same time period.

### Protocol violations

**Figure 6:**
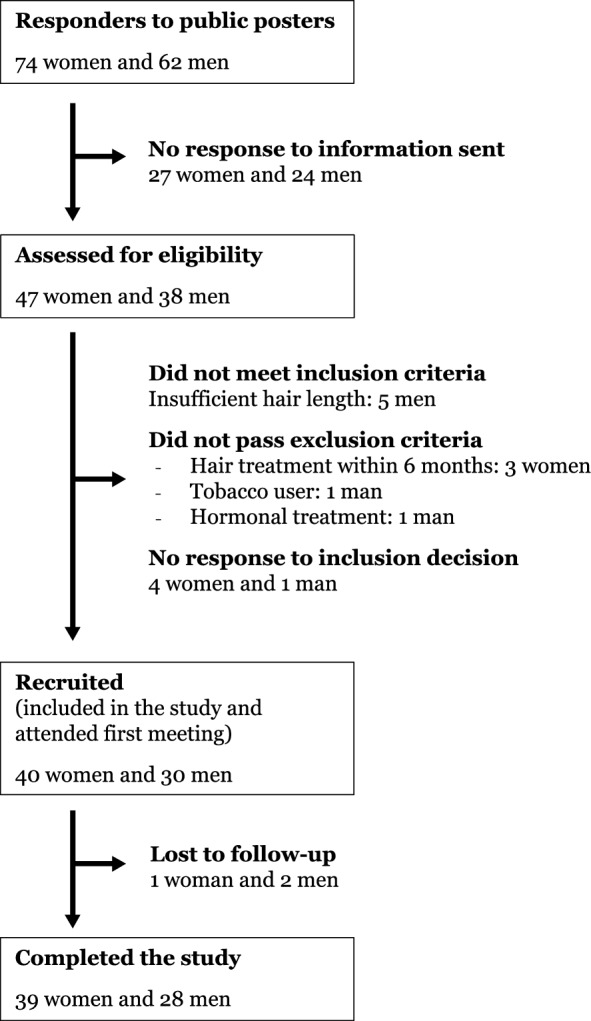
Flow chart of participants.

Two men and one woman were lost to follow-up and did not provide any information on the reasons for not completing the study, see Fig. 6. One male participant lost all his saliva samples. Thus, hair from the second sampling was available for 28 men and 39 women, and saliva samples were available from 27 men and 39 women.

## Discussion

This observational study evaluated the potential of hair testosterone measurements in general and segmental hair analysis as a retrospective testosterone diary in particular. Testosterone patterns along the hair were preserved over time to some degree, but concentrations were generally higher more distally from the scalp. Testosterone concentrations in hair were correlated to testosterone concentrations in saliva and could on a group level reliably differentiate between men and women. In addition to sex, also hair colour and relationship status were associated with hair testosterone levels. Finally, the intra-individual variation in hair outgrowth over a three-month period identified a rarely considered confounder in the field of hormonal hair analysis.

To our knowledge, only one previous study has compared testosterone concentrations (washed in isopropanol and analysed with an immunoassay) in outgrown hair segments with previously sampled scalp-near segments. The correlation coefficients ranged from r = 0.58 (*p* = 0.099) to r = 0.88 (*p* = 0.002), no data on the segmental testosterone concentrations was available^17^. Although we could demonstrate a preserved relation in concentrations between adjacent segments as the hair grew, the absolute concentrations were higher more distally from the scalp. A possible explanation could be continuous incorporation of hormones not only in the hair follicle but also more distally, for example through contact with sebum. Sebaceous glands and sweat glands contain androgen-synthesizing enzymes and androgens influence the production of sebum, but whether the sebum itself contains hormones has, to our knowledge, not been studied^18^. In hair from pigs and cattle, Otten et al.^19^ described how distal hair segments show greater permeability compared to proximal hair segments to external contamination with urine, leading to markedly increased cortisol concentrations in contaminated hair. Likewise, repeated immersion in water resulted in loss of cortisol in distal hair segments compared to proximal segments. This is also in line with our finding that increased hair wash frequency moderated the distal increase in testosterone concentrations, with the possible rationale being that testosterone from sebum and/or cortex is washed away and that the effect of hair washing has a cumulative effect in the more distal hair segments. Consequently, it is plausible that the previously described decreases in hair cortisol concentrations in distal hair segments, when a pre-extraction wash has been used, leads to increased removal of lipophilic substances from distal hair segments compared to scalp-near hair. Another possibility is that hair matrix degradation could affect the ratio between the total sample weight and the hormone content. In vitro hair washing and exposure to visible light and UV light has been shown to damage hair, reducing the tensile strength of the hair and damaging the protective cuticle^20–22^. Increased photodamage in distal hair segments compared to proximal segments was described by Cao et al.^23^ and the same pattern of damage could be reproduced by exposing hair from the proximal segment to increasing amounts of UV light.

Validation of testosterone analysis in hair through correlation with salivary testosterone concentrations has only been attempted a few times with contradicting results^17,24^. The current results showed that mean salivary testosterone and testosterone in hair correlated significantly. As mentioned above, the local production of steroid hormones in the skin, independent from the hypothalamo–pituitary–gonadal axis, could potentially blur the relationship between the two matrices^25,26^. Also, methodological differences such as different antibodies in the assays used for the hair and saliva analyses, respectively, could add some variance. Further, a recent study on radiolabelled cortisol administered to rhesus monkeys describes how cortisol was not only incorporated in hair in its native form but more readily incorporated in the form of cortisone as well as other yet unidentified radiolabelled metabolites in the hair samples^27^. Perhaps a true measure of mean retrospective concentrations of the unbound testosterone hormone fraction in serum should include testosterone metabolites in the hair, which needs to be explored in future research. Interestingly, hair testosterone analysis demonstrated a superior ability to discriminate between the sexes compared to mean salivary testosterone, which underpins the physiological relevance of the testosterone levels in hair. Hair washing frequency differed slightly between the sexes (women: mean 2.68 times per week, SD 1.18; men: mean 4.00 times per week, SD 2.05), with more frequent hair washing habits among men, meaning that the difference in hair testosterone concentrations between sexes could potentially be even more distinct if the analysis had been corrected for hair washing frequency. The salivary analysis was calculated with a mean value of 16 saliva samples per individual, which should correct for the intra-day and inter-day variation in salivary testosterone concentrations. A possible explanation to the superior discrimination between sexes of hair testosterone are that the time frame for saliva sampling in the current study may have been suboptimal and did not capture the time during the day when the difference between the sexes would have been the most pronounced.

Regarding the relation between background variables and testosterone in hair, the regression analysis showed significant effects for sex and natural hair colour, as well as significantly lower hair testosterone levels in individuals cohabitating with a partner compared to singles or persons not cohabitating with a partner, adjusted for potential age differences between the groups. To our knowledge, only Voegel et al. have evaluated testosterone concentrations in hair with regard to pigmentation, where no intra-individual difference between pigmented and grey hair was found (n = 18). Several studies focusing on cortisol have addressed the issue of natural hair colour, with ambiguous results. Higher cortisol concentrations in individuals with black hair compared to individuals with lighter shades has been reported^28–30^, as well as no differences in hair cortisol concentrations in relation to natural hair colour^7^. It has also been hypothesised that differences in hair hormone concentrations across categories of hair colour would be an effect of ethnicity^28,31^. During year 2020 19.7% of the population in Sweden was either born abroad or had parents that both were born abroad^32^. Unfortunately, there was no information collected on the ethnical background of our study participants. Ad hoc, we performed Spearman correlation on mean salivary testosterone with the different hair colour shades as an ordinal variable (men and women apart), and found no significant correlations between salivary testosterone and natural hair colour (rho = −0.122 to 0.117, *p* = 0.473 to 0.970). This could support the effect of hair pigmentation specifically on the hair testosterone concentrations. Regarding possible matrix degradation, the melanin pigment counteracts cortical damage from visible light and UV light, with the least photobleaching occurring in black hair^33,34^. In an in vitro setting, irradiating a hydrocortisone solution and intact hair strands has been shown to decrease hydrocortisone concentrations in the solution as well as decrease the cortisol concentrations in hair^8^. Interestingly, in the same study by Grass et al*.* dehydroepiandrosterone and progesterone did not have the same pattern of decreasing concentrations as cortisol.

The current study confirms previous research regarding an average scalp hair growth rate of about 10 mm per month. Growth rate has generally been studied in regrown hairs previously shaved or clipped, with a follow up of a few days to a fortnight, which also allowed for identification of growing and resting hair follicles at a certain time-point^35,36^. With a longer follow up time, of weeks to months, the impact of hair follicles switching between growing, transitioning and resting phases (anagen, catagen and telogen) on the total hair growth should be detectable and influence the individual lengths of regrown hairs within a sampling area. Hair follicles transition between growth phases independently of each other, the proportion of growing hair follicles and mean growth rate varies during the year and towards the end of the anagen phase the growth has been shown to gradually slow down^37,38^. This has the potential to influence the temporal relationship between a certain hair segment and a specific time period in the past^39^. Descriptions of intra- and inter-individual variability in hair growth rate have been surprisingly scarce, given its important implications for hair hormone analyses. To the best of our knowledge, a within-individual variation in hair growth rate impacting the length of regrown hairs during a few months has previously not been described. This finding highlights a confounder that is not commonly considered in hormonal hair analysis, hampering the temporal resolution when attempting to use hair as a retrospective hormone diary.

## Limitations

Based on our previous study the sample size of the current study (n = 66) should suffice to reliably detect a difference between the sexes^13^. Using repeated sampling and multiple observations per person increases the quality of the present data. But, to evaluate the contribution of background variables to hair testosterone concentrations such as BMI, educational level or hair heat treatments per week, the number of observations were not sufficient. A bigger cohort is needed to ensure a greater generalisability of, in particular, the regression analysis. Also, the sparseness of published research on hair testosterone analysis limits the possibilities to assess the plausibility of our results, in particular regarding the segmentally increasing testosterone concentrations as well as the variability in hair growth rate.

## Conclusions

The results from this study demonstrate that there is a degree of segmental hormone conservation over time, but also highlight potential confounders. Firstly, testosterone concentrations increased with distance from the scalp, factors contributing to this segmental increase need to be identified in future research. Secondly, hair wash frequency seems to affect hair hormone concentrations, but chiefly in distal hair segments. In general, hair testosterone analysis needs to be adjusted for sex and the natural hair colour. Interestingly, lower hair testosterone levels are found in individuals with stable relationships (cohabitating with their partner) in an age-adjusted regression. And lastly, intra- and inter-individual variability in hair growth rate influences the temporal resolution along the hair. The current results indicate that repeated hair sampling near the hair roots during a study may be preferable to analysing concentration changes between proximal and distal segments within the same hair sample.

## Data Availability

The data collected and analysed during the current study are not publicly available due to the private nature of the data, but are available from the corresponding author on request.

## Abbreviations

BMI: Body mass index
ELISA: Enzyme-linked immunosorbent assay
EQF: The European qualifications framework
ROC: Receiver operating characteristic
UV: Ultra violet
